# Agonist anti-4-1BB plus neutralizing anti-CTLA-4 or -PD-L1 synergize to promote tumor regression by rescuing dying dysfunctional CD8+ T cells within the tumor microenvironment

**DOI:** 10.1186/2051-1426-3-S2-O10

**Published:** 2015-11-04

**Authors:** Brendan Horton, Jason B Williams, Stefani Spranger, Thomas F Gajewski

**Affiliations:** 1 University of Chicago, Chicago, IL, USA

## 

Tumors can be broadly placed into two categories: those that are T cell-inflamed and those that are not, with T cell-inflamed tumors having specific chemokine and type I interferon gene expression signatures as well as CD8^+^ tumor-infiltrating T cells (TIL). The fact that these T cell-inflamed tumors are not destroyed by the CD8^+^ TIL argues that mechanisms in the tumor microenvironment must render the CD8^+^ TIL dysfunctional. In agreement with this argument, baseline infiltration of CD8^+^ T cells appears to be necessary for tumor regression in response to neutralizing antibodies against CTLA-4 and PD-L1, indicating that these immunotherapies work predominantly on T cells already infiltrating the tumor microenvironment. We analyzed CD8^+^ TIL in the mouse B16.SIY model of melanoma to identify additional immunotherapy targets that might synergize with blockade of CTLA-4 and the PD-1/PD-L1 pathway. Using knowledge from in vitro T cell anergy gene expression profiling and phenotypic analysis, we found that dysfunctional CD8^+^ TIL unexpectedly express several co-stimulatory molecules, including 4-1BB. We therefore investigated whether delivering positive signals through co-stimulatory receptors, in combination with blockade of specific inhibitory receptors, could act on dysfunctional TIL to restore their function and induce tumor regression. We found that an agonist anti 4-1BB antibody synergized with inhibitory antibodies against either CTLA-4 or PD-L1 to induce tumor regression of established B16.SIY tumors. By using the S1P1 inhibitor FTY720 to block T cell egress from lymph nodes, we found that these combinations did not require newly primed cells from the periphery for tumor regression, arguing that they work directly in the tumor microenvironment. Deeper analysis of TIL revealed expansion of SIY-reactive CD8+ TIL and restored IL-2 production, as well as decreased expression of inhibitory receptors PD-1, LAG-3 and 2B4. Investigation of the mechanism of increased TIL number revealed that proliferation of the TIL was not enhanced with these combinations. Rather, a marked decrease in apoptosis of SIY-reactive TIL was observed, as measured by decreased active caspase-3 levels during tumor regression induced by 4-1BB combination immunotherapy. 4-1BB may therefore provide critical survival signals that rescue dying dysfunctional CD8^+^ cells in the tumor microenvironment, allowing them to be re-functionalized by CTLA-4 or PD-L1 blockade to mediate tumor regression.

